# Hyperglycemia-Induced Changes in Hyaluronan Contribute to Impaired Skin Wound Healing in Diabetes: Review and Perspective

**DOI:** 10.1155/2015/701738

**Published:** 2015-09-10

**Authors:** Sajina Shakya, Yan Wang, Judith A. Mack, Edward V. Maytin

**Affiliations:** ^1^Department of Biomedical Engineering, Lerner Research Institute, Cleveland Clinic, 9500 Euclid Avenue, Cleveland, OH 44195, USA; ^2^Department of Dermatology, Dermatology & Plastic Surgery Institute, Cleveland Clinic, 9500 Euclid Avenue, Cleveland, OH 44195, USA

## Abstract

Ulcers and chronic wounds are a particularly common problem in diabetics and are associated with hyperglycemia. In this targeted review, we summarize evidence suggesting that defective wound healing in diabetics is causally linked, at least in part, to hyperglycemia-induced changes in the status of hyaluronan (HA) that resides in the pericellular coat (glycocalyx) of endothelial cells of small cutaneous blood vessels. Potential mechanisms through which exposure to high glucose levels causes a loss of the glycocalyx on the endothelium and accelerates the recruitment of leukocytes, creating a proinflammatory environment, are discussed in detail. Hyperglycemia also affects other cells in the immediate perivascular area, including pericytes and smooth muscle cells, through exposure to increased cytokine levels and through glucose elevations in the interstitial fluid. Possible roles of newly recognized, cross-linked forms of HA, and interactions of a major HA receptor (CD44) with cytokine/growth factor receptors during hyperglycemia, are also discussed.

## 1. Introduction

Diabetes, which affects ~24 million people or 8% of the U.S. population [1], is a disease in which abnormal glucose metabolism plays a central role. Hyperglycemia (defined as high glucose levels in the bloodstream) results from loss of pancreatic insulin-producing cells in type 1 diabetes, or loss of normal insulin-responsiveness of target cells such as muscle and fat cells in type 2 diabetes, and leads to severe clinical complications over time [2]. These complications include renal failure, retinopathy, atherosclerosis, peripheral vascular disease, loss of peripheral sensory nerve function (neuropathy), and impaired wound-healing ability. The combined effects of neuropathy and poor wound healing lead to formation of nonhealing skin ulcers of the feet and lower limbs, a particularly common problem in diabetics. Nearly 15% of diabetic individuals will develop a foot ulcer, and many eventually lose a limb to amputation, at an overall healthcare cost of at least $10.9 billion per year in the U.S. [3]. Recent evidence suggests that impaired wound healing in diabetic patients is directly related to poorly regulated serum glucose levels. For example, a recent clinical study in diabetics showed that high levels of hemoglobin A1c, an indicator of poor hyperglycemic control, correlate directly with delayed wound healing [4]. Unfortunately, despite improved methods for providing proper insulin delivery and glucose control, nonhealing wounds and chronic ulcers remain a persistent problem.

Hyaluronan (HA) is a glycosaminoglycan, present in all mammalian tissues, that is composed entirely of two sugar subunits, glucuronic acid and N-acetylglucosamine [5]. Very abundant in skin and many other tissues, HA is structurally simple yet functionally complex [6]. Here, we review evidence that impairments in wound healing in diabetics are mechanistically linked to hyperglycemia through altered synthesis and degradation of hyaluronan (HA). After providing a short wound healing-orientation, we will summarize evidence from the literature showing that hyperglycemia leads to degradation of HA in the glycocalyx (pericellular coat) of endothelial cells, thereby increasing leukocyte recruitment and creating a proinflammatory microenvironment that adversely affects not only blood vessel function, but also adjacent pericytes, smooth muscle cells, and fibroblasts.

In the skin, inflammation and fibrosis (scar formation) are prominent features during normal wound healing, as reviewed in more detail in this HA special issue [7]. Wound healing is a very complex process involving three overlapping phases that comprise the* inflammatory phase*, ~days 1–3;* tissue regenerative (proliferative) phase*, ~days 3–10; and* remodeling phase*, ~2 weeks to 3 months [8, 9]. On days 1–3, a wave of neutrophils is recruited from the bloodstream. Responding to local cytokine gradients, the neutrophils adhere to the luminal wall of small postcapillary venules, traverse the endothelium, and migrate into the dermal tissue. On day 3, a second wave of leukocytes (monocyte/macrophages) arrive to engulf and remove the dead neutrophils that have released their granules and undergone apoptosis. Both neutrophils and macrophages secrete large amounts of cytokines including transforming growth factor beta (TGF-*β*), the major cytokine that stimulates fibroblasts to differentiate into activated, contractile myofibroblasts. Myofibroblasts are the cells that produce most of the collagen and other matrix molecules that comprise the new extracellular matrix (ECM), or scar. Scar tissue is gradually remodeled over many months, approaching but never quite reproducing the original architecture; thus, healed adult wounds typically attain only ~70% of the tensile breaking strength of unwounded skin. The overall sequence of events during wound healing implies that inflammation and fibrosis are closely linked. Excessive or prolonged presence of inflammatory cells in a wound will drive myofibroblast conversion, increase fibrosis (with imperfect macromolecular assembly of collagen and GAG elements), and result in functionally impaired scar tissue.

In diabetes, wound healing is delayed [10]. A major reason for this appears to be chronic, enhanced inflammation, which disrupts the timing of ECM synthesis and ultimately reduces the quality of the restored collagen architecture. Diabetic patients with poor hyperglycemic control appear to be in a heightened inflammatory state, even in the absence of wounding or infection. This important concept was convincingly demonstrated by a clinical study in which 108 diabetic patients and 36 healthy controls were followed prospectively for ~2 years [11]. At study initiation, patients were ulcer-free, but foot ulceration developed in nearly one-third (29%) of the diabetic patients over time. At the first study visit, all patients underwent blood sampling and forearm skin biopsy for measurements of serum protein markers and skin histopathology. Remarkably, the levels of many cytokines and proteins associated with inflammation (interleukins IL-6 and IL-8; tumor necrosis factor alpha, TNF-*α*; C-reactive protein; fibrinogen) were significantly elevated in the diabetic patients at baseline. In biopsies from forearm (unwounded) skin, diabetics had a higher number of cells overall, and the number of inflammatory cells around blood vessels (a strong indicator of inflammation) was higher. The diabetic subjects also had higher expression of CD31 (in proliferating vessels) and MMP-9 (in leukocytes and vessels) in the skin. These findings suggest a preexisting, hyperinflammatory state. Experimental studies in diabetic animal models, including mice [12] and rabbits [13], have also clearly demonstrated baseline increases in inflammatory cytokines (IL-6, IL-8, and TNF-*α*) in diabetic skin in the absence of wounding.

We propose that this chronic, proinflammatory state in diabetic skin may be caused by underlying abnormalities in HA and other GAGs that form the pericellular matrix on the apical surfaces of the endothelial cells (EC). The pericellular matrix (glycocalyx) is a structure, comprising HA and various GAG-containing proteoglycans, that is present to varying degrees on most cell types [14]. In Sections 2 and 3 below, we describe data showing that the glycocalyx on the luminal surface of EC in blood vessels is altered (reduced) as a result of hyperglycemia and discuss mechanisms for how this might lead to proinflammatory consequences. In Section 4, we speculate on how hyperglycemia and proinflammatory cytokine signaling may affect perivascular cells, changing the balance between cell proliferation and cell death and altering the production of ECM.

## 2. Hyperglycemia Leads to Loss of the Endothelial Cell Glycocalyx in Small Vessels of the Skin

The structure and importance of the endothelial glycocalyx have been described in several reviews [14–16]. Located on the luminal side of blood vessel walls, this glycocalyx is an important structure with several critical functions, including the following: (1) serving as a reservoir for antithrombotic factors such as antithrombin III that prevent clotting and as a shield to prevent direct contact between circulating platelets and the EC which would trigger further clot formation; (2) regulating the production of nitric oxide (NO) and NO-mediated signaling in response to flow-induced shear stress; (3) modulating the permeability of macromolecules at tight junctions between endothelial cells; (4) modulating leukocyte adhesion.

A recent critical discovery is that* in response to hyperglycemia, the thickness of the glycocalyx on blood vessel endothelia is significantly reduced*, leading to loss of protective functions and other deleterious changes. In studies in humans, the thickness of the endothelial glycocalyx can be measured indirectly by comparing intravascular distribution volume of a glycocalyx permeable tracer (e.g., dextran 40) with that of an impermeable tracer (e.g., labeled erythrocytes). Nieuwdorp et al. showed that acute hyperglycemia in healthy subjects was associated with a ~50% reduction in glycocalyx volume [17]. Reduction of the glycocalyx was likely due to damage by reactive oxygen species (ROS) generated under hyperglycemic conditions, because infusion of the antioxidant N-acetylcysteine (NAC) could prevent the reduction [17]. To test this hypothesis further, glycocalyx measurements were performed in type 1 diabetic patients and in age-matched controls [18]. In addition to the indirect tracer method used above, orthogonal polarization spectral (OPS) imaging was also employed to visualize the sublingual microcirculation (blood vessels beneath the tongue). Both the systemic glycocalyx volume and the directly measured glycocalyx thickness were reduced by 50–86% in type 1 diabetic patients relative to normal controls [18]. The same research group showed that HA is an important component of the glycocalyx and is affected in diabetes [19]. They did this by measuring the activity of hyaluronidases, HA-degrading enzymes that comprise five members in mammals (HYAL1, HYAL2, HYAL3, HYAL4, and PH20; HYAL1 and HYAL2 are the major hyaluronidases in somatic tissues) [20]. Hyaluronidase activity and circulating levels of HA were both elevated in the serum of diabetic patients [19], suggesting that hyaluronidase activity may contribute to the degradation of the endothelial glycocalyx in diabetes.

### 2.1. Studies on Atherosclerosis Provide Evidence That the EC Glycocalyx Is Important for Understanding Diabetic Vascular Abnormalities

Nieuwdorp et al. showed that perturbation of HA metabolism predisposes patients with type 1 diabetes mellitus to atherosclerosis [19]. These investigators performed a study to evaluate the relationship between HA and hyaluronidase with carotid intima-media thickness (cIMT), an established surrogate marker for cardiovascular disease. In type 1 diabetic patients and control subjects, the cIMT of patients' carotid arteries was measured, and multivariate regression analyses were performed to determine relationships between HA, hyaluronidase, and cIMT. Type 1 diabetics showed structural changes of the arterial wall associated with increased HA catabolism, thereby supporting altered glycosaminoglycan metabolism in type 1 diabetes as a potential mechanism involved in accelerated atherogenesis.

Hyaluronidase was also the subject of a study by Kucur et al. [21], in which 162 nondiabetic patients undergoing coronary angiography, with or without stable coronary artery disease (CAD), were examined. Hyaluronidase assays in these patients showed significantly increased serum hyaluronidase activity in patients with CAD, suggesting a role of HA degradation in the pathophysiology of atherosclerosis.

Another study examined the potential role of HA in the development of atherosclerosis by using an inhibitor of HA synthesis (4-methylumbelliferone) in atherosclerosis-prone mice [22]. That study, discussed more fully in Section 3.4, concluded that inhibition of HA synthesis interferes with the protective function of the glycocalyx and accelerates atherosclerotic progression.

In addition to HA, the glycocalyx of ECs also contains proteoglycans, comprising heparan sulfate (HS) and chondroitin sulfate (CS) chains linked to core proteins such as syndecan and versican, and all interact with HA either directly or indirectly; see the review by Reitsma et al. for a concise summary of these GAG components [14]. The importance of HS in the endothelial glycocalyx was suggested by a study in which heparanase (an enzyme that degrades HS) was microinfused into capillaries of the hamster cremaster muscle, leading to an increase in the concentration of red blood cells flowing within those capillaries [23]. If the heparanase was heat-inactivated prior to infusion, then the effect was abolished. These data suggested that HS in the endothelial glycocalyx is important for physiological responsiveness of the capillary. A study by Han et al. extended this to diabetes, by showing that decreased amounts of HS-containing proteoglycans (HSPGs) can contribute to diabetic endothelial injury [24]. Porcine aortic ECs were cultured in normal glucose (5.5 mM), or in high glucose (30 mM), in the presence or absence of insulin. The concentrations of GAGs associated with cells or free in the culture media were determined. High glucose decreased the amount of GAGs associated with cells and increased GAGs in the medium; the addition of insulin to high glucose cultures restored the levels of GAGs toward normal. Thus, hyperglycemia induces HSPG degradation or inhibits its synthesis (likely due to EC injury), and insulin may have a direct protective effect on the endothelium, independent of its blood glucose-lowering effect.

### 2.2. Hyperglycemia Decreases Normal Endothelial Cell (EC) Functioning and May Lead to Lethal EC Damage

Risso et al. examined human umbilical vein endothelial cells (HUVECs) in culture and showed that intermittent high glucose enhances apoptotic cell death [25]. HUVECs cultured for 14 days in media containing glucose at 5 mM, 20 mM, or a daily alternating regimen of 5 or 20 mM showed more apoptosis in the cells exposed to intermittent high glucose. In the study, levels of Bcl-2 (apoptosis-protective) and Bax (apoptosis-promoting) proteins were examined. Continuous hyperglycemia led to a relative decrease in Bcl-2 and an increase in Bax levels; these changes were larger in magnitude during intermittent high glucose exposure. Thus, in diabetics, variability in glycemic control could be more deleterious to the endothelium than constant high glucose concentrations.

Another study, by Zuurbier et al, reinforces the idea that intermittent hyperglycemia is just as bad, if not worse, than continuous glucose elevation. Mice that were normoglycemic, transiently hyperglycemic (25 mM) for 60 min due to glucose infusion, or hyperglycemic (25 mM) for 2–4 weeks (db/db mice) were studied [26]. The glycocalyx was probed using a 40 kDa Texas Red dextran (control) known to permeate the glycocalyx under all conditions, along with 70 kDa FITC dextran which is excluded from the glycocalyx in healthy animals. Clearance of these dyes from the blood was measured. Short-term hyperglycemia caused a rapid decrease in the ability of the glycocalyx to exclude 70 kDa dextran, interpreted as a loss of barrier function of the glycocalyx; the barrier loss was just as severe as that observed with continuous hyperglycemia.

## 3. Potential Molecular Mechanisms for How HA May Regulate Vascular Inflammation in Diabetes

We will now discuss, in more detail, proposed molecular mechanisms that may link altered HA in the endothelial glycocalyx to inflammation and vascular complications of diabetes.

### 3.1. Inflammation and Thrombosis in Vessels Are Exacerbated by Platelet-Derived Hyaluronidase

The EC glycocalyx is a reservoir for various antithrombotic factors, such as antithrombin III, and a reduction in this storage pool due to thinning of the glycocalyx in diabetes could alter the balance between pro- and anticoagulatory factors [14]. Furthermore, a reduced glycocalyx volume could encourage interactions between platelets and endothelial cell-surface receptors and promote platelet adhesion and aggregation. Also, platelets are known to promote leukocyte recruitment and adhesion, further enhancing the inflammatory milieu [27]. Platelets were recently discovered to mediate another proinflammatory mechanism, one involving HA cleavage. In 2009, de la Motte et al. demonstrated that human platelets contain exclusively HYAL2, but no HYAL1 (whereas most human cells contain both). HYAL2 is a hyaluronidase that is GPI-anchored on the cell membrane and cleaves extracellular HA into intermediate-sized fragments, whereas lysosomal HYAL1 degrades HA into very small fragments [28]. Platelet HYAL2 is stored in *α*-granules and upon platelet activation becomes expressed on the cell surface, where it functions to degrade extracellular HA matrix [29]. The HA fragments generated by platelet-derived HYAL2 initiate inflammatory and angiogenic signaling by stimulating mononuclear leukocytes in the immediate microenvironment to produce proinflammatory cytokines, such as IL-6 and IL-8 [28]. The critical take-home message here is that when intact HA is degraded, smaller fragments are formed that are highly angiogenic, inflammatory, and immunogenic in a size-dependent manner [30, 31] and can trigger cellular stress responses (by serving as endogenous danger signals) [32, 33].

### 3.2. Loss of the EC Glycocalyx in Diabetes Leads to Reduced Nitric Oxide (NO) Production

Nitric oxide (NO) is a nonpolar gaseous molecule that is synthesized and released by endothelial cells and subsequently acts upon vascular smooth muscle cells to reduce vascular tone [34]. NO is synthesized by NO synthases (NOS). ECs express the endothelial form of NOS (eNOS), which can be activated by several factors including flow-related shear stress activation of stretch-operated nonselective cation channels (SOCC) in the cell membrane, leading to elevation of intracellular calcium levels, activation of eNOS, and the production of NO from L-arginine and molecular oxygen [35]. Other mechanisms by which shear stress may activate eNOS have been proposed, including phosphorylation by protein kinases associated with the intracellular cytoskeleton or through interaction with membrane receptors such as CD44 that are colocalized within lipid rafts along with other signaling molecules including eNOS [36–38].

As described in Section 2, the EC glycocalyx is reduced by hyperglycemia. Thus, one might expect that a loss of glycocalyx could affect shear-induced activation of eNOS, NO synthesis, and vasodilation, and indeed this seems to be the case. When canine femoral arteries were treated with hyaluronidase to degrade the HA glycocalyx, shear-induced NO production was impaired [39]. Heparitinase treatment also impaired NO production in bovine EC, showing that disruption of heparan sulfate in the glycocalyx could also affect NO production [40]. In fact, removal of each of the three different specific components of the glycocalyx (HA, heparan sulfate, and sialic acid, but* not* chondroitin sulfate) was shown to significantly reduce shear-induced NO production in bovine ECs [38].

Other studies have shown that the impairment of shear-induced NO responses observed during hyperglycemia involves the cytoskeleton. Studying porcine aortic EC in a parallel flow chamber, Kemeny et al. showed that hyperglycemia impairs intracellular actin alignment and NOS activation in response to shear stress [41]. Under normal glucose conditions, the alignment of EC cytoskeletal actin fibers increased by 10% in response to shear, but in high glucose, actin fiber rearrangement increased by less than 3%. eNOS phosphorylation increased with shear stress for ECs in normal glucose but did not significantly change for cells in high glucose [41]. Chen et al. used atomic force microscopy (AFM) and laser scanning confocal microscopy (LSCM) to study the role of cytoskeletal rearrangement and NO synthesis in HUVECs [42]. Under constant high glucose (25 mM) or fluctuating high glucose (25 mM/5 mM), HUVECs released significantly less NO into the culture supernatants; the cells also exhibited decreased eNOS expression and showed mechanical stiffening of the cell membrane. A NOS enzyme inhibitor (L-NAME) elicited similar effects. Overall, the conclusion from these two studies is that hyperglycemia is associated with stiffening of endothelial cell membranes, altered cytoskeletal alignment, and decreased NO synthesis in response to flow shear stress, all of which could result in stiffer blood vessels and a reduction in vasodilatory capacity, thereby predisposing vessels to increased risk of occlusion and thrombosis.

Hypothetically, loss of HA in the EC glycocalyx during hyperglycemia could reduce shear stress-induced NO production via at least two mechanisms. HA normally binds to CD44, a receptor that resides in membrane lipid rafts that also contain eNOS, and perhaps other signaling molecules that may be necessary for eNOS activation [37]. A loss of HA:CD44 interaction might therefore reduce local eNOS activity. Alternatively, CD44 is known to interact with the cytoskeleton (via the cytoplasmic tail of CD44, described more in Section 3.3), so that theoretically a reduction in HA:CD44 signaling could affect cytoskeleton-associated protein kinases that phosphorylate eNOS and thus reduce NO production.

### 3.3. Hyperglycemia Impairs the Ability of the EC Glycocalyx to Regulate the Permeability of Macromolecules

The EC glycocalyx influences blood flow and presents a selective barrier to movement of macromolecules from plasma to the endothelial surface. Work by Henry and Duling advanced the notion that permeability of the endothelial glycocalyx is determined by HA [43]. Video microscopy was used to visualize microvessels in the hamster cremaster muscle, to investigate whether HA was involved in permeation properties of the glycocalyx (i.e., the ability of tracer molecules to penetrate into the apical glycocalyx). The glycocalyx thickness was visualized as a clear space between the anatomic diameter of a small blood vessel and the diameter of red blood cells within that vessel. The investigators asked whether several fluorescently tagged dextran tracers (70, 145, 580, and 2000 kDa), all normally too large to enter the glycocalyx, might do so after experimental degradation of HA. After a 1 h treatment with hylauronidase, increased access of the 70 and 145 kDa dextran tracers into the glycocalyx was observed (i.e., hyaluronidase appeared to create a more open matrix, enabling the smaller dextran molecules to penetrate deeper into the glycocalyx). Following hyaluronidase treatment, an infusion of a mixture of high molecular weight (MW) HA and chondroitin sulfate was able to reconstitute the glycocalyx. Thus, HA appears to be an essential component for glycocalyx barrier function.

In inflamed blood vessels, leakage of fluid and protein occurs through gaps between the ECs. Singleton et al. noted that this leakage is increased in diabetes, and they proposed a mechanism in which interactions between glycocalyx HA and CD44, a major cell-surface receptor for hyaluronan, may be involved [44, 45]. CD44, “floating” in the cell membrane, is localized in cholesterol-rich microdomains called lipid rafts, which are important regulators of vascular integrity [46]. Normally, full-length HA is bound to the extracellular portion of CD44; this ligand-receptor attachment promotes interactions between the internal (cytoplasmic) residues of CD44 and signaling molecules such as Akt/Tiam1/Rac1 and Annexin A2/protein S100, which then enhance the formation of cortical actin filaments that strengthen intercellular contacts between neighboring ECs [44, 45]. Hyperglycemia reduces the amount of HA in the glycocalyx (e.g., through degradation by hyaluronidase), leaving less HA available to bind to CD44. Loss of HA:CD44 binding reduces downstream CD44 signaling. This leads to loss of cortical actin filaments (through conversion to actin stress fibers) and permits EC to pull apart, now allowing leakage of fluid and protein to occur at intercellular junctions and thus contributing to the inflammatory microenvironment.

### 3.4. Changes in the EC Glycocalyx during Hyperglycemia Promote Leukocyte Adhesion

First, we will review evidence that hyperglycemic injury can lead to increased recruitment of leukocytes at sites of activated (inflamed) blood vessels in the skin. We will then discuss some current ideas about how HA may be involved in this process.

Since the reference point for studies on diabetes should always be the patient, we mention again the clinical report by Dinh et al. (discussed in the Introduction) that reported higher numbers of perivascular leukocytes associated with small cutaneous blood vessels in diabetic patients, relative to normoglycemic controls [11]. However, without experimental data, it is not possible to predict how HA specifically may affect leukocyte adhesion. For example, work by the Siegelman group in the late 1990s established that the expression of HA on cultured ECs is increased by exposure to proinflammatory cytokines (TNF*α* and IL-1*β*) and that this HA induction is selective to ECs derived from microvasculature and not from large vessels [47]. Furthermore, lymphocytes were shown to tether and adhere to this HA glycocalyx via their CD44 receptors [48]. From these observations, one might predict that a reduced HA glycocalyx during hyperglycemia should lead to less rather than more leukocyte adhesion, whereas in most wound-healing studies the opposite is observed. Since the majority of leukocytes found in early wounds are neutrophils and macrophages, not lymphocytes, different recruitment mechanisms amongst different leukocyte subtypes remain a distinct possibility.

To begin to investigate mechanisms for the role of the EC glycocalyx in leukocyte recruitment in an animal model in vivo, Constantinescu et al. employed venules of the mouse cremaster muscle to test the hypothesis that glycocalyx degradation stimulates leukocyte-endothelial cell adhesion [49]. When heparitinase was administered locally to degrade the endothelial glycocalyx, an increase in adherent leukocytes was observed. Another agent that degrades the endothelial glycocalyx is oxidized low density lipoprotein (Ox-LDL); when Ox-LDL was administered systemically into the mouse, leukocyte adhesion increased dose-dependently. Perfusion of the venules with heparan sulfate or heparin reconstituted the endothelial glycocalyx and attenuated the Ox-LDL-induced leukocyte-endothelial cell adhesion. The authors concluded that circulating heparan sulfate and heparin attach to the venule wall and attenuate Ox-LDL-induced leukocyte immobilization.

Nagy et al. examined the potential role of HA synthesis in the development of atherosclerosis in a mouse model [22]. This study in Apo E deficient mice addressed the effects of long-term pharmacological inhibition of HA synthesis on vascular function and atherosclerosis. Administration of 4-methylumbelliferone (4-MU), an inhibitor of HA synthesis, led to a marked increase of aortic plaque burden at 25 weeks. An increase in recruitment of macrophages to vascular lesions was detected after 2 and 21 weeks of 4-MU treatment. Also, severe damage of the endothelial glycocalyx after 2 and 21 weeks of 4-MU was detected by electron microscopy of the innominate artery and myocardial capillaries. The authors concluded that systemic inhibition of hyaluronan synthesis by 4-MU interferes with the protective function of the endothelial glycocalyx, thereby facilitating leukocyte adhesion, subsequent inflammation, and progression of atherosclerosis.

**Figure 1:**
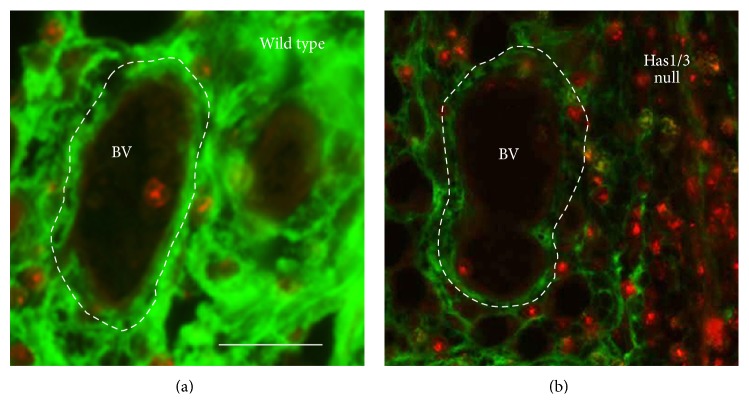
Skin biopsies from a wound edge at 24 h after wounding. Specimens from (a) normal mice (*wild type*) or (b) knockout mice lacking the genes for HAS1 and HAS3 (*Has1/3 null*) were stained with an HA binding probe to visualize HA (green) and an anti-myeloperoxidase antibody to visualize neutrophils (red). Note the marked loss of HA and increase in neutrophils extravasating from the blood vessel (BV) in the HAS1/3 deficient skin. Bar, 50 *μ*m.* From [50], used with permission*.

In our lab, we have utilized HAS1/HAS3 double-knockout (HAS1/3 null) mice as another model in which to study effects of altered HA upon leukocyte recruitment from the vasculature. In mammals, three HA synthase enzymes (HAS1, HAS2, and HAS3) are normally expressed. Deletion of the* Has2* gene is embryonic lethal in mice, due to defects in cardiac development; however, mice lacking* Has1* and/or* Has3* genes are normal and viable [50]. Interestingly, cutaneous wound healing is abnormal in the HAS1/3 null mice and is associated with increased wound inflammation due to enhanced recruitment of neutrophils at day 1 and macrophages at day 3, emanating from the cutaneous microvasculature at the wound site [50]. Compared to the normal amounts of HA in the blood vessels and surrounding dermis in wild type mouse skin (Figure 1(a)), reduced amounts of HA in the dermis and blood vessel walls (including HA associated with the EC) are observed in HAS1/3 null mice in the same vessels at which large numbers of neutrophils appear to be recruited (Figure 1(b)). Further experiments are ongoing in our laboratory to test the hypothesis that reduced HA in the endothelial glycocalyx allows leukocytes greater access to adhesion molecules such as V-CAM and I-CAM on the endothelial surface.

### 3.5. Changes in HA Structure Play a Role in Promoting Leukocyte Adhesion

Up to now, we have discussed how HA is* quantitatively* altered during hyperglycemia; that is, its amount is reduced in the glycocalyx. However, HA can also undergo* qualitative* changes, in terms of altered structural properties, when it is synthesized by cells undergoing a stress response such as exposure to high glucose. As summarized in a recent mini review [52], cells exposed to a variety of stressful conditions produce a form of HA that is leukocyte-adhesive. This HA is “sticky,” forming aggregates in cell culture that resemble thick HA “cables” [53, 54]. Aggregation is due, at least in part, to cross-linking of HA strands via protein subunits (the heavy chains of inter-alpha-trypsin inhibitor) that are added covalently through the enzymatic action of tumor necrosis factor-stimulated gene 6 protein, TSG-6 [55–58].

Because HA is produced from intracellular glucose-derived precursors (glucuronic acid and N-acetylglucosamine), HA synthesis appears to be particularly sensitive to local concentrations of glucose in the tissue. Recent work by Wang and Hascall in renal mesangial cells showed that an important cellular mechanism for dealing with high glucose (e.g., 25 mM; normal is ~5 mM) is to make an aggregated form of HA (“HA cable” structure) that reflects abnormal production of HA inside the cells [52]. HA is normally made by HAS enzymes at the plasma membrane and extruded directly into the extracellular space, whereas HA produced during hyperglycemia is synthesized within intracellular compartments by HAS enzymes residing in the ER, Golgi apparatus, and transport vesicles. This internal HA is then eliminated from the cell through an autophagy mechanism in which HA-containing autophagosomes fuse with the plasma membrane and then release the aggregated form of HA into the extracellular environment. The aggregated HA cables are then removed by macrophages.

The teleological concept here is that HA serves as a kind of “glucose trap” during times of excessive cytosolic glucose influx, helping cells to eliminate glucose by incorporating it into a stable polymer via internal synthesis of HA within endoplasmic reticulum (ER) and Golgi membranes, which is then released into the extracellular space as HA cables [52]. However, elimination of internal HA comes at a price, because the HA cables are highly adhesive for leukocytes that can trigger inflammation and cause tissue destruction. This hyperglycemia-induced mechanism for producing proinflammatory HA matrices is now well established for mesangial cells of the diabetic kidney [59, 60] and more recently for adipose cells exposed to high glucose [61]. An interesting question that we plan to investigate is whether a similar autophagic HA mechanism might be operative in the EC glycocalyx of small blood vessels in diabetic skin.

## 4. Potential Molecular Mechanisms by Which HA, Altered in Diabetes, May Regulate Fibrosis

### 4.1. Diabetes and the Abnormal Production of ECM by Cells Surrounding the Blood Vessels

Glucose exchange across capillary walls is known to occur by diffusion through pores between the endothelial cells (reviewed in [62]). This free exchange is the basis for the development of new devices for continuous glucose monitoring which allow one to estimate plasma glucose concentrations from measurements of glucose concentration in the interstitial fluid of skin [63–66]. In diabetics [62, 64] and in healthy volunteers [67], interstitial glucose concentrations in the dermis reach nearly the same levels as glucose in the plasma, although with a well-described time lag in uptake kinetics. This time lag, while important for clinical glucose monitoring, is not important for the biology under consideration here, where it is sufficient to know that cells in extravascular locations (in the dermis adjacent to the blood vessels) are intermittently exposed to high glucose concentrations and undergo hyperglycemic stress. These extravascular cells include fibroblasts, smooth muscle cells, and pericytes.

Pericytes are a particularly interesting type of cell, being very closely associated with small cutaneous blood vessels and having cytoplasmic processes that wrap around the circumference of capillaries and postcapillary venules, making contact with the ECs [68]. While relatively little is known about them, pericytes appear to play an important supportive role for EC proliferation and survival. Indeed, the loss of pericytes by apoptosis in the eye correlates with loss of retinal ECs in diabetic individuals and is a major mechanism leading to retinopathy and blindness [69]. In the skin, 3D reconstructions of electron microscopic images of capillaries showed that pericytes in diabetic skin are still present but exhibit an abnormal morphology that consists of hypertrophy, abnormal cytoplasmic branching, and gaps in basement membranes that probably account for the known increase in vascular permeability [68]. Interestingly, the walls of these vessels are thickened by deposition of a belt of basement membrane-like material admixed with variable amounts of collagen fibrils, along with another unidentified material deposited within the vascular wall itself [68, 70]. These classical studies, while limited to morphological observation, strongly suggest that the pericyte (a mesenchymal cell type) produces increased amounts of abnormal ECM that contributes to a sclerotic or fibrotic vascular phenotype in diabetics and impairs the proper functioning of the vessel. From our earlier considerations, we postulate that increased levels of proinflammatory cytokines, exposure to high glucose levels, or both are contributing to abnormal pericyte morphology and excessive production of ECM.

### 4.2. Hyperglycemia Promotes Fibrosis by Increasing HA Synthesis and Decreasing Apoptosis in Smooth Muscle Cells and Fibroblasts

**Figure 2:**
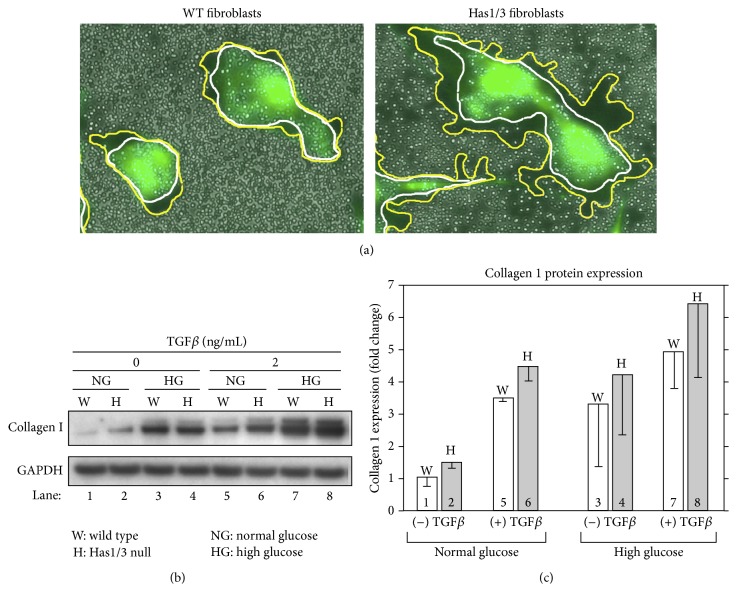
Skin fibroblasts with a larger HA glycocalyx are more responsive to TGF-*β* and produce more collagen when cultured in a high glucose medium. Skin fibroblasts from either wild type (WT) or Has1/3 null mice were cultured in normal glucose (1 g/L) or in high glucose (4.5 g/L) in the absence or presence of TGF-*β*1 (2 ng/mL) in DMEM media with 1% FBS for 48 hours. (a) Erythrocyte exclusion assay to visualize the pericellular coat on WT and Has1/3 null fibroblasts [51]. Cells were labeled with calcein to delineate the cell bodies. The HA glycocalyx is indicated between the white and yellow lines. (b) Western blot of protein from WT or Has1/3 null fibroblasts, probed with an antibody to type 1 collagen. GAPDH, loading control. (c) Quantification of protein band intensities from two independent western analysis experiments, performed under the conditions shown in panel (b) and normalized to GAPDH.* White bars*, WT fibroblasts.* Gray bars*, Has1/3 null fibroblasts.* Error bars*, mean ± half-range.

HA biology can be surprisingly cell-specific. Thus, while hyperglycemia causes problems in dermal microvessels by reducing HA in the EC glycocalyx, hyperglycemia affects dermal mesenchymal cells by increasing their HA glycocalyces. Work from the Tammi group showed that, in vascular smooth muscle cells (SMCs), high glucose promotes HA synthesis and HAS gene expression but impairs vascular SMC function as reflected by a reduced ability to contract collagen gels; this offers a potential mechanism whereby hyperglycemia could disturb vascular remodeling and contribute to arteriosclerosis [71]. Vigetti et al. showed that HMW-HA can protect human aortic SMCs against apoptosis induced by 4-methylumbelliferone (an HA synthesis inhibitor), suggesting that higher amounts of HA associated with SMCs increase their survival and might favor accumulation of SMCs in the vessel wall [72]. A similar relationship in which higher HA production appears to be protective against apoptosis was demonstrated in our studies of dermal fibroblasts [51]. Using HAS1/HAS3 double-knockout mice [51], we showed that fibroblasts compensate for the loss of HAS1 and HAS3 by overexpressing HAS2, resulting in an increase in HAS2 activity and synthesis of an enlarged HA glycocalyx relative to normal fibroblasts (Figure 2(a)). When exposed to apoptosis-inducing stress such as serum starvation, the HAS1/3 null cells were less likely to die [51].

We recently began to investigate how these murine fibroblasts respond to hyperglycemia. Elegant studies by the Steadman group in human lung fibroblasts have shown that CD44 (a major HA receptor) interacts with the TGF-*β* receptor (TGF*β*R) and that this interaction is obligatory for full activity of downstream TGF-*β* signaling, including the conversion to myofibroblasts [73, 74]. Inhibition of HA abrogates TGF-*β* pathway activity in their system [73, 74]. We hypothesize that a similar mechanism may apply to murine skin fibroblasts. Figures 2(b) and 2(c) provide preliminary evidence to support this idea. In fibroblasts from HAS1/3 null mice (i.e., cells with large HA coats), collagen-synthetic responses are elevated relative to wild type fibroblasts under the following conditions: at baseline in normal glucose (lane 2); in response to 2 ng/mL of TGF-*β*1 in normal glucose (lane 6); and in response to the combination of high glucose and TGF-*β*1 (lane 8). These observations are consistent with the notion that the HA glycocalyx supports TGF*β*R-driven profibrotic processes. Work in our laboratory is ongoing to test this hypothesis further.

## 5. Conclusion

**Figure 3:**
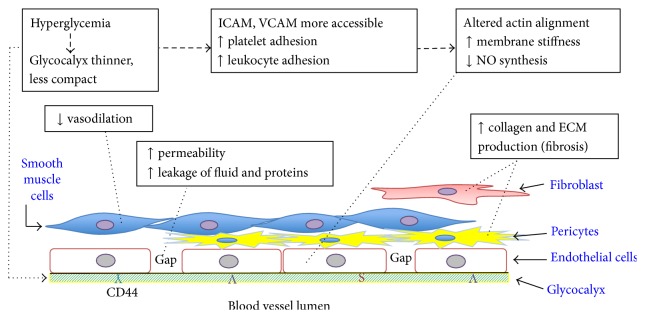
Summary of hyperglycemia-induced changes, within blood vessels and in their immediate surroundings, that involve alterations in HA.

In this review, we have discussed current evidence regarding mechanisms by which altered hyaluronan (HA) in microvessels of the skin may lead to poor wound healing in diabetic patients. These ideas are summarized in Figure 3. The major premise is that hyperglycemia causes loss of the HA-containing glycocalyx on endothelial surfaces of cutaneous microvessels, leading to increased leukocyte adhesiveness and triggering the release of cytokines that contribute to a proinflammatory environment. Such changes are likely to increase oxidative damage, decrease proper responsiveness of vascular ECs, and impede neoangiogenesis and other repair responses during the tissue regenerative phase of wound healing. Other cell types at or near the blood vessel wall, including pericytes, smooth muscle cells, and fibroblasts, are also adversely affected by hyperglycemia and the cytokine-rich environment but through different mechanisms. One mechanism may be an enhanced sensitivity to profibrotic cytokines such as TGF-*β*, whose receptors interact with CD44 and with HA in the glycocalyx of the mesenchymal cells. A better understanding of hyperglycemia-related mechanisms in the skin is a worthy goal, as it may suggest new approaches to heal or prevent skin ulcers in patients with diabetes.
